# Transition from hospital to nursing home: Discharge planners as a potential lever for quality improvements?

**DOI:** 10.1007/s00391-024-02325-0

**Published:** 2024-07-17

**Authors:** Kristina Kast, Lukas Carl

**Affiliations:** 1 Department of Healthcare Management, Friedrich-Alexander-Universität Erlangen-Nürnberg (FAU), Lange Gasse 20, 90403 Nürnberg, Germany; 2 Medical Valley EMN e. V., Henkestraße 91, 91052 Erlangen, Germany

**Keywords:** Public reporting, Discharge management, Quality of care, Online survey, Quality representation agreement, Qualitätsdarstellungsvereinbarung, Entlassmanagement, Versorgungsqualität, Online-Befragung, Qualitätsdarstellungsvereinbarung

## Abstract

**Background:**

Public reporting is supposed to be helpful in differentiating between well and poorly performing nursing homes; however, hospital patients often have difficulties to deal with quality information. Discharge planners (DP) can support them in comparing quality and, by influencing patients’ decision, lead to better provision of care in nursing homes.

**Objective:**

This study investigated the choice behavior of DP, their use of quality information and the potential to impact the decision-making of patients.

**Material and methods:**

A total of 70 DP from German hospitals with a geriatric department participated in an online survey. They were asked about information preferences and tools used for nursing home searches. In addition, they assessed quality information items from the new German quality reporting on a Likert scale. To test their comprehension participants were given a case scenario of a typical patient, were shown nursing homes displayed based on a medical comparison portal navigator (AOK-Pflegenavigator) and were asked to select nursing homes in a 3-round experiment.

**Results:**

When looking for a nursing home, DP primarily rely on internal nursing home directories (*n* = 62; 92.5%). The 3 preferred criteria for decision are: distance to the family (*n* = 55; 28.80%), bed availability (*n* = 51; 26.7%) and wishes of patients/relatives (*n* = 41; 21.47%). The consent score for public reporting was 46.28% and the comprehension ratio was 82.24%.

**Discussion:**

The DP do not advise hospital patients on the performance of nursing homes and rely on the decision-making of patients. This results in a lack of impact on patients’ decisions and consequently in a loss of potential for public reporting to lead to better care in nursing homes.

**Supplementary Information:**

The online version of this article (10.1007/s00391-024-02325-0) contains supplementary material, which is available to authorized users.

## Abstract (de)

**Hintergrund:**

Öffentliche Qualitätsberichterstattung soll eine Differenzierung zwischen guten und schlechten Pflegeheimen ermöglichen. Krankenhauspatienten sind jedoch mit Qualitätsvergleichen oft überfordert. Entlassmanagementpersonal (EMP) kann ihnen dabei helfen und so zu Qualitätsverbesserungen im Pflegeheimen beitragen.

**Ziel:**

Diese Studie untersucht das Wahlverhalten des EMP, seinen Umgang mit Qualitätsinformationen und das Potenzial, die Entscheidung Betroffener zu beeinflussen.

**Material und Methoden:**

EMP (*n* = 70) aus deutschen Krankenhäusern mit geriatrischer Fachabteilung nahm an einer Online-Befragung teil. Es wurden Informationspräferenzen und die Tools erfragt, die das EMP bei der Pflegeheimsuche verwendet. Zusätzlich bewertete das EMP auf einer 5‑Punkte-Skala verschiedene Informationen aus der neuen Qualitätsberichterstattung in der Pflege. In einem Verständnistest hat das EMP, basierend auf einem Patientenszenario, in 3 Runden Pflegeheime gewählt, deren Darstellung auf dem medizinischen Vergleichsportal AOK-Pflegenavigator basierte.

**Ergebnisse:**

Bei der Pflegeheimsuche verlässt sich das EMP hauptsächlich auf interne Pflegeheimverzeichnisse (*n* = 62; 92,5 %). Entscheidungen basieren auf 3 zentralen Kriterien: Entfernung des Heims von Angehörigen (*n* = 55; 28,80 %), Bettenverfügbarkeit (*n* = 51; 26,70 %) und Wünsche der Betroffenen (*n* = 41; 21,47 %). Die Zustimmungsquote für die neue Qualitätsberichterstattung lag bei 46,28 % und die Verständnisquote bei 82,24 %.

**Diskussion:**

EMP handelt pragmatisch und verlässt sich auf die Entscheidung der Betroffenen. Somit findet keine umfangreiche Unterstützung bei Qualitätsvergleichen statt und das Potenzial, die Entscheidung Betroffener zu beeinflussen und so zu Qualitätsverbesserungen beizutragen, geht verloren.

## Introduction

Publishing provider quality information on medical comparison portals (MCP) can help consumers of long-term care services to compare between well and poorly performing nursing homes. The selection of good- over bad-performing providers can trigger quality improvements in the healthcare system [3]; however, older hospital patients and their relatives might have difficulties in dealing with quality data [14]. Discharge planners (DP) “may be in the best position to translate publicly available information about [nursing homes] quality to [patients] and their families” [2].

## Background

### Quality reporting

The MCP are a web-based instrument of public reporting [19]. Public reporting is a method of making healthcare quality information transparent to consumers [25]. It is supposed to enable consumers to assess the performance of health services providers, so they can select the best [7]. The choices made by consumers (also called “selection pathway”) pressure nursing homes to undertake improvement measures [3], which facilitates better care delivery in the long run [25].

Historically, public reporting started with the communication of mortality rates in the hospital sector in the USA and the UK. Other countries followed these examples and implemented public reporting by creating numerous MCP [19]. Germany implemented the mandatory public reporting on long-term care services in 2008 [13]. The agencies responsible for quality checks are the medical boards that conduct inspections in nursing homes approved for statutory health insurance [32]. Until 2019 the inspection results included criteria, such as *care of residents with dementia*, *everyday life support*. These results were displayed as school grades in transparency reports on the internet [4]; however, this system has been heavily criticized [34] and has now been replaced by the new Quality Representation Agreement, which led to changes in the information type and presentation.

According to this agreement, the new public reporting is based on 3 mandatory pillars [13]. First, nursing homes have to provide facility-related information (e.g., *equipment*, *specializations*). Second, the facilities have to undergo annual external inspections conducted by the Medical Boards. The inspections results include information, such as *supporting residents with personal hygiene*, or *wound treatment*. The third pillar consists of quality indicators from the internal quality management conducted by the facilities themselves. This pillar includes information, such as *pressure ulcers* or *serious falls*. Instead of school grades, this information is now displayed in the form of boxes and circles. [5]. The collected data from these pillars go to a central unit called the data clearing office, which analyzes it for publishing on MCP like AOK-Pflegenavigator (AOK care navigator).

### State of research

Some literature investigating the impact of public reporting on healthcare quality concluded that the “selection pathway” regularly fails to work effectively (e.g., [20]); however, when assessing the effectiveness of public reporting, it should be taken into account that we are dealing with a complex intervention consisting of many components which all together have to work perfectly to achieve an impact [26]. Thus, like any other technology, public reporting needs further research efforts on how it can be improved [22]. As Sandmeyer and Fraser stated, it is important to investigate “when, how, and why does it work—and for whom?” [26]. We follow this request and focus on an area in healthcare that forms an important and rarely studied interface between hospitals and nursing homes. At this stage of care, patients referred to a nursing home are dependent on the support by DP who can impact their decision.

Until now, few studies investigated on the role of DP in the transitional process connected with public reporting. In a US study, Shugarman and Brown asked DP about information preferences [29]. Collier and Harrington additionally asked about tools DP used for nursing home searches in the USA [8]. Both studies were conducted two decades ago. We assume that the increased importance of public reporting and digitalization efforts in healthcare systems have changed the underlying processes analyzed in these studies. We conducted a study that, beyond the information preferences and search behavior, investigated the attitude of DP toward the new public reporting, introduced as a consequence of the Quality Representation Agreement in Germany.

### Discharge planning

According to § 11 (4) of the German Social Code, Book Five, by discharge from hospital to home or to other care settings, hospital patients are entitled to receive a comprehensive discharge planning including medical transfer services [33]. The latter concentrates on the transition to other facilities like nursing homes and could be expanded to the step of admission into the hospital [21]. Discharge planning involves patients and relatives as well as the multidisciplinary work of different professionals like physicians, nurses, therapists and pharmacists [16]. These parties are coordinated by a DP of the hospital. Usually, DP are trained hospital nurses or additional staff in the hospital, often holding a nursing qualification or qualified in social work [11]. The DP are also referred to as social service workers, transitional care workers, case managers, or discharge managers [23]. There is little uniformity to the structuring of the discharge concept across hospitals [10]. Depending on the discharge concept of the hospital and the qualification of the DP their tasks vary greatly but always encompass the discharge preparation.

## Aims, objectives and research questions

To explore the potential impact of German DP on the decision of hospital patients when comparing quality, our objectives were to explore how DP choose a nursing home for a patient and to examine their attitude toward new public reporting. Consequently, we addressed the questions: (1) What instruments do DP use? (2) What information do DP use during decision making? (3) How important is the information from the new public reporting for them? (4) Do they understand the new public reporting?

## Study design and investigation methods

### Sample choice

**Fig. 1:**
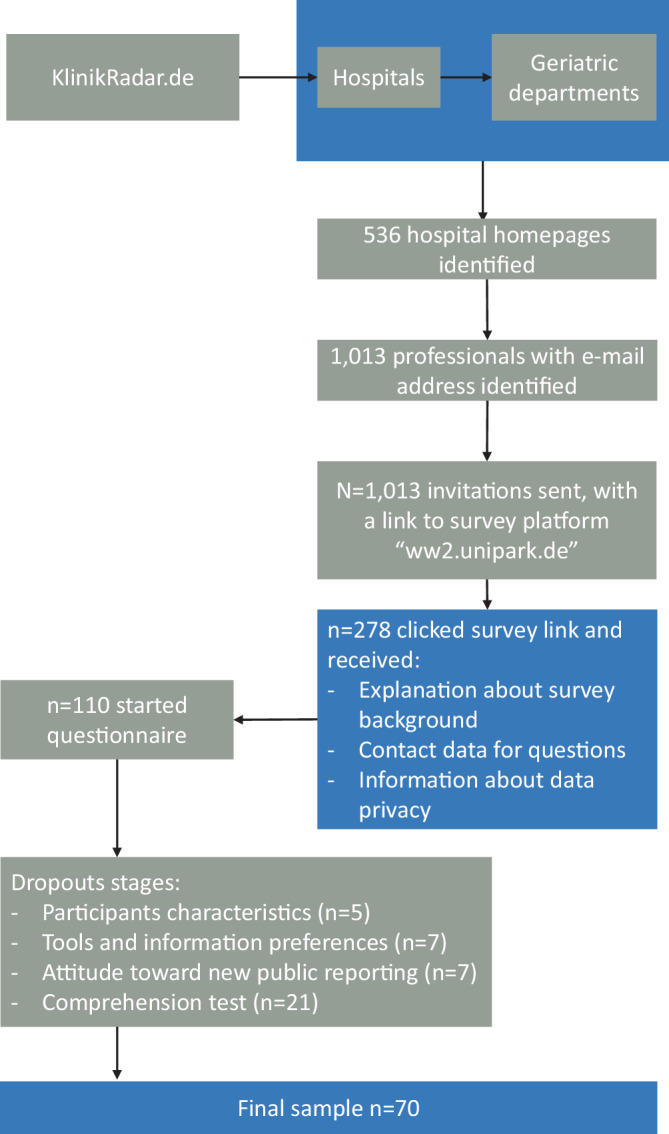
Illustration of sampling process stages

We recruited DP for an online survey on the platform unipark.de. We chose all professionals who worked in discharge management departments of hospitals (social service workers, discharge managers, case managers, or transitional care workers) with geriatric departments as a suitable population. We reached out to 1013 professionals via e‑mail which contained a short description and a link to the online survey. We had a net response rate of 6.91%. The complete process is shown in Fig. 1.

### Data collection

**Fig. 2:**
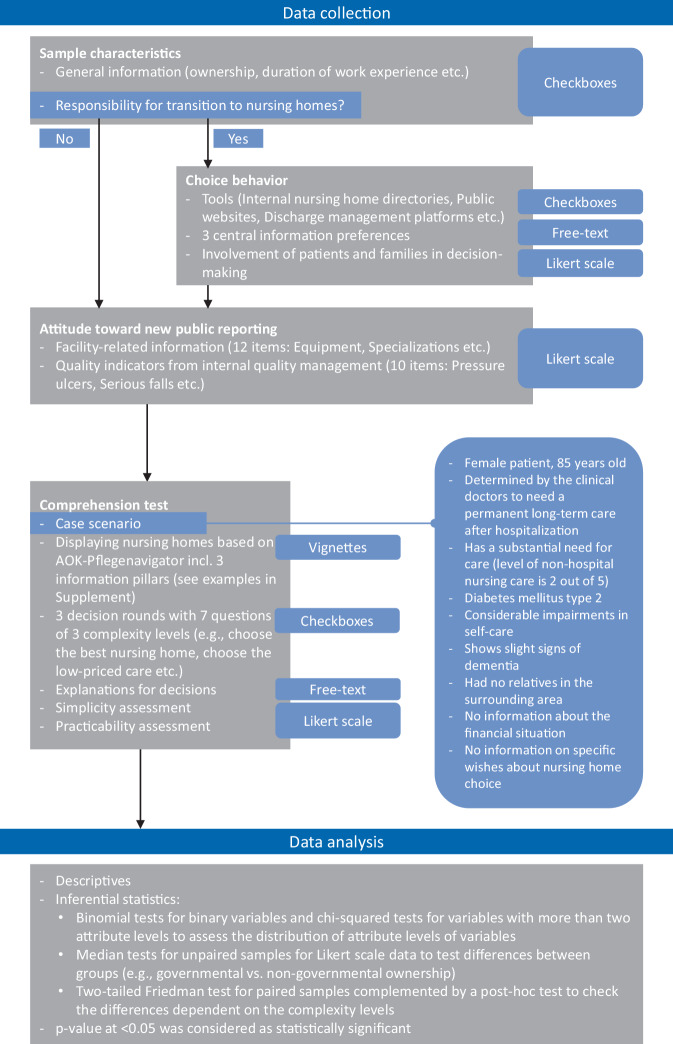
Illustration of data collection and analysis

We pretested the questionnaire between 1 April 2022 and 8 April 2022. The final data collection was conducted between 18 April 2022 and 31 May 2022 and is shown in Fig. 2. Participants were informed on data privacy in the invitation and before survey start. First, they were asked questions on their characteristics, later, we asked about the tools used and their information preferences when searching a nursing home for a patient. In addition, we displayed 22 information items from the new German public reporting and asked to assess them on a Likert scale for their importance in a decision-making process. To test their comprehension, participants were presented with a case scenario based on data from the annual nursing care report [15] simulating a typical patient. In 3 consecutive rounds, we displayed 3–4 available nursing homes at a time. The visualization was derived from the MCP AOK-Pflegenavigator (AOK care navigator) and included information from the 3 pillars of the new public reporting (examples in Supplement Fig. S1). Participants were asked to make decisions of different complexity levels (e.g., level 1 choose the best nursing home) as previous studies reported that the comprehension can be dependent on information complexity (e.g., [27]). The survey participation was voluntary and anonymous and did not include patients. On average, participants needed 17:42 min to complete the questionnaire.

### Data analysis

Answers given as free text were first synthesized inductively by grouping similar statements into categories. After that, we calculated descriptives for all variables. For the analysis of the perceived importance of the 22 information items, we calculated consent scores by weighting the scale with 0 (“not important”) to 100 (“very important”) before calculating mean values. In the comprehension test, every correct answer was considered as 1 point achieved and summarized to a comprehension ratio with the highest possible score of 7 points. Inferential statistics were also calculated when appropriate and are concretized in Fig. 2 and in the respective figures and tables in the text. All data were analyzed with the software IBM SPSS Statistics 26, Armonk, NY: IBM Corp.

## Results

### Sample

A total of 70 participants completed the survey. Their characteristics are shown in Table 1. Most of them (*n* = 59; 84.3%) were social service workers with a maximum of 10 years of experience (*n* = 40; 57.1%). They mostly worked in hospitals with a public ownership (*n* = 33; 50.8%) or in not-for-profit hospitals (*n* = 20; 30.8%).

**Table 1:** Study sample overview

Variables	Characteristics	Count	%
*Profession*	Social service worker	59	84.3
	Transitional care worker	5	7.1
	Case manager	3	4.3
	Discharge manager	3	4.3
*Work experience (in years)*	0–10	40	57.1
	11–20	14	20.0
	> 20	16	22.9
*Leading position*	Yes	21	30.0
	No	49	70.0
*Ownership*	Governmental	33	47.1
	Nonprofit	20	28.6
	Private	12	17.1
	Not specified	5	7.1
*Transfer responsibility*	Yes	67	95.7
	No	3	4.3
Total sample: *n* = 70			

### Choosing a nursing home for a patient

**Fig. 3:**
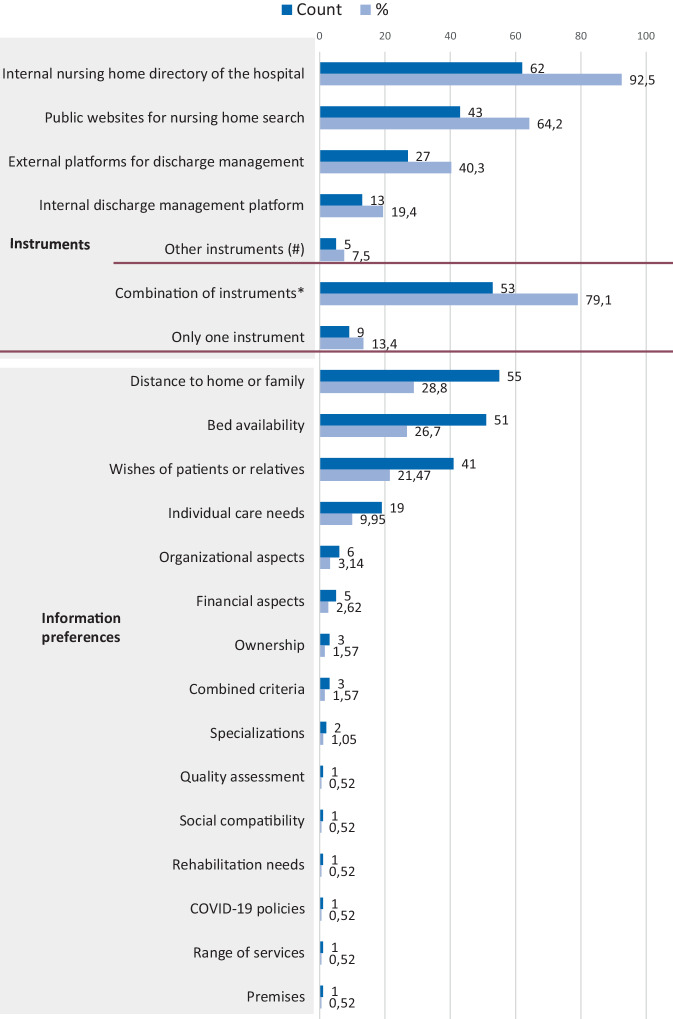
Results for choice behavior. *n* = 67. ^a^other instruments: cooperation, care support centers, updates by nursing homes, telephone calls, clinical network. *p < 0.001, binomial test

*Internal nursing home directories* are the most frequently used instrument (*n* = 62; 92.5%) when searching for a facility (Fig. 3 and Supplement Table S1). Many participants (*n* = 43; 64.2%) also use MCP for nursing home search. *External* (*n* = 27; 40.3%) or *internal discharge management platforms* (*n* = 13; 19.4%) are used less often. Some participants (*n* = 5; 7.5%) stated that they use other instruments than the options given in the survey. For example, they make a telephone call to the cooperating nursing homes. Out of 70 participants 79.1% (*n* = 53) selected more than only 1 instrument (*n* = 9; 13.4%). The most frequently stated combinations were *internal nursing home directories* with MCP (*n* = 39; 55.7%) and *internal nursing home directories* with *external platforms for discharge management* (*n* = 25; 35.7%). The 3 central decision criteria were the *distance to home or family* (*n* = 55; 28.80%), *bed availability* (*n* = 51; 26.70%), and *wishes of patients/relatives* (*n* = 41; 21.47%).

### Attitude toward public reporting

**Fig. 4:**
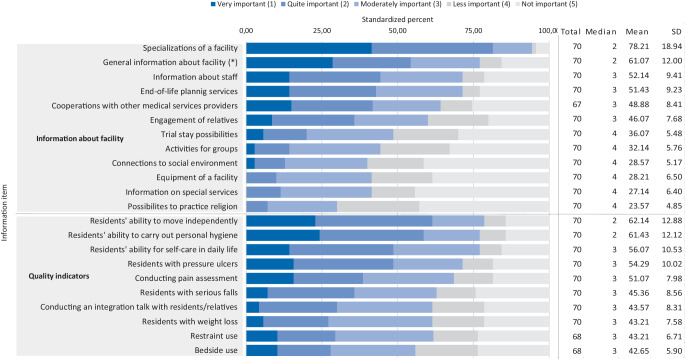
Assessment of information from new public reporting. Overall mean: 46.28%; *p = 0.009, median test for differences between participants from governmental (med = 3) and non-governmental (med = 2) hospitals. *med* median; *SD* standard deviation

The overall consent score for publicly available quality information was 46.28% (Fig. 4). *Specializations* and *general information* were two facility-related information items rated as “important” (med = 2). Another 4 items (e.g., *engagement*) were rated as “moderately important” (med = 3) and 6 (e.g., *equipment*) received low priority (med = 4). In terms of quality indicators, participants rated information on *residents’ ability to move independently* and *to carry out personal hygiene* as most important (med = 2). The remaining information items (e.g., *pressure ulcers*) were rated as “moderately important” (med = 3).

**Fig. 5:**
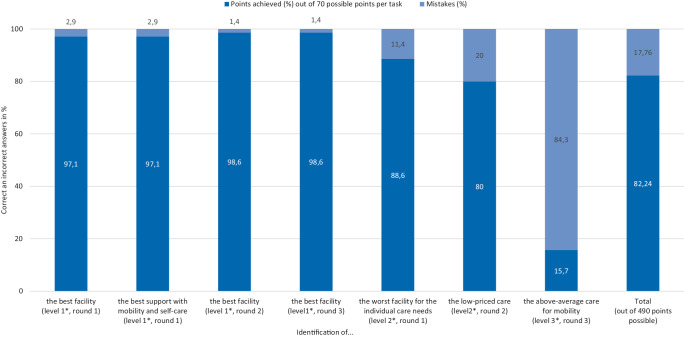
Comprehension test results. *p = 0.000 for every complexity level, Friedman test with post hoc test

The overall comprehension ratio on quality information was 82.24% (Fig. 5 and Supplement Table S2). When asked to identify “the best” facility (level 1), participants made few mistakes (*n* = 2; 2.86%; *p* = 0.000). The more complex tasks like the identification of “the worst” facility resulted in more mistakes (*n* = 8; 11.4%; *p* = 0.000). The most wrong assessments (*n* = 59; 84.3%; *p* = 0.000) occurred at level 3 where participants had to identify multiple facilities with above-average care based on quality indicators.

## Discussion

### Consequences for patients

Our results suggest that German DP act pragmatically when choosing a nursing home for a hospital patient. In our study, the dominant search instrument were *internal nursing home directories*, which is similar to the US study of Collier and Harrington who’s participants relied on information booklets and marketing materials [8]. Also, in line with this study, our participants based their decisions mainly on general conditions. For example, free beds were with 26.7% one of the 3 central decision criteria in our study while Collier and Harrington also reported their participants “were primarily concerned about bed availability” [8]. Another pattern that emerged in both studies is that DP completely relied on the decision of others. For example, 1 of the 3 central criteria in our study were *wishes of patients and relatives* (21.47%); in Collier and Harrington DP also focused on patient and/or family preferences (46%) or recommendations of physicians (51%) [8]. These circumstances may be the reason why in our, but also in the studies of Collier and Harrington [8] and Shugarman and Brown [29] *distance to home or family* was the criterion of the highest relevance.

In our study, the DP did not recognize the value of public reporting. The consent score was low at 46.28%. Furthermore, when showing information items based on AOK-Pflegenavigator, they were more interested in *general information about facility* rather than “hard facts” like *pressure ulcers*. Some participants explained this with being “critical of these inspection procedures.” Mistrust in quality information can be a serious barrier for considering MCP [18]. It is worth noting however that in the comprehension test, the patient in the case scenario did not have any specific wishes and no relatives in the surrounding area, resulting in the participants stronger reliance on other criteria (e.g., *quality assessment* with *n* = 44 statements, Supplement Fig. S2) than before the test. As one DP said: “Since [the patient] has no relatives who would visit her daily and she has a considerable impairment in everyday activities, the information on *support in organizing everyday life and social contacts* as well as on *support in special care situations* (because of her diabetes) were crucial for my decision”.

Our study shows that DP do not see themselves as a consultant, but rather as an executing agent and the fact that a patient has relatives seems to discourage them from undertaking the decision responsibility and to choose based on quality. To strongly consider the wishes of affected people is a noble approach and can be understood as a part of shared decision making. It might however impede the selection of a good nursing home. Research has shown that in particular low educated patients are at risk to be transitioned to a low-quality facility [2]. Moreover, when families make decisions, it does not result in higher satisfaction [31]. Thus, to help patients/relatives to find a high-quality nursing home, they should be more actively advised [24].

### Consequences for quality improvements

The literature suggests that the “selection pathway” fails to be effective because consumers do not include public reporting in their decision making [12]. Not understanding quality information [9] or having difficulties to use it for decision making [27] are possible reasons for ignoring quality information. In contrast to these explanations, our study suggests that DP are able to understand the information provided on MCP (82.24% comprehension score). Furthermore, most participants (*n* = 53, Supplement Fig. S3 and Fig. S4) in our study felt that the new quality reporting was convenient to use and aided their decision making. Another possible reason for not using quality information is not being aware of it [6]. This is also not supported by our findings, which show that using MCP by DP is more common today (60.2%) than two decades ago (24% [8]). Thus, DP can be assumed to have a high potential in having an impact on patients’ decisions and helping them to act according to the “selection pathway”; however, not feeling responsible for consultation, mistrusting the information and not seeing a value in public reporting indicate that DP use MCP for pragmatic reasons but not to comparing quality. Taken together, our findings suggest that current choice behavior and the attitude of DP toward the public reporting result in a loss of potential to have an impact on patients’ decisions when comparing quality of nursing homes.

For the future, we should be aware of one more issue. As Shugarman and Garland highlighted in their study, time constraints are also one of the barriers for considering quality information by DP [30]. In their work routines, German DP are also faced with severe time constraints [1, 28]. Thus, even if we succeed in sensitizing DP for their role as a consultant, they probably will be unable to integrate this task into their busy schedule. To enable them to adequately support patients and relatives, we need better system regulations which unburden DP. Furthermore, there are already actors in the market, offering to assist with discharge planning at no cost to the hospital. In return, these actors try to monetize their connections to patients by offering newly needed services to patients [21]. As these activities might hamper informed decision-making of patients, transparently integrated solutions are more desirable for the healthcare system. A recent German study investigated the transition from hospital to home [17] via the help of external visit nurses who get involved in the discharge planning early and assist until patient’s situation in the next setting is stable. This concept could be worth considering in the transition from hospital to nursing home to make the “selection pathway” work.

### Limitations

The comprehension ratio of our sample was high but it also worsened with higher complexity levels. Thus, we see the high comprehension ratio as an indicator for good information understanding and consequently a high potential of DP; however, the actual situation on MCP is more complex, and the comprehension should be tested separately in an experiment that better reflects this complexity. Moreover, our argument that DP more intensively react on public reporting if patients have no relatives should also be considered as an assumption as it was a side effect of our study, and not chosen to be analyzed beforehand. This assumption could be tested in a specific experiment.

## Conclusions and practical recommendations

We found no substantial changes in the choice behavior of DP compared to prior studies. Our results suggest that even if DP understand the new public reporting well, because of mistrust they see no value in it and refrain from informing patients and relatives on nursing home performances. They see themselves as executing agents who rely on decision-making of others. This lack of impact on patients’ decisions results in a loss of potential for the “selection pathway” to lead to better care in nursing homes.

To improve the situation, the following changes are needed:

- DP should be sensitized for their role and potential in the process.
- Time capacities of DP should be expanded via better system regulations.
- Supporting DP with external transitional care services integrated in the healthcare system can help.

## Supplementary Information

Additional information on methodology and results of the study.
