# Supplementary material for: Transition from hospital to nursing home: Discharge planners as a potential lever for quality improvements?
Source: Z Gerontol Geriatr. 2024 Jul 17;57(8):631–8. doi: 10.1007/s00391-024-02325-0 (PMC11602860; doi:10.1007/s00391-024-02325-0)
Supplement: Supplementary file 1 — Additional information on methodology and results of the study. [file 391_2024_2325_MOESM1_ESM.pdf]

Supplementary material for the manuscript “**Transition from hospital to nursing home: Discharge planners as a potential lever for quality improvements?**”

Authors: Kristina Kast, Lukas Carl

DOI: 10.1007/s00391-024-02325-0

Fig. S1 Examples of formats shown in the comprehension test in different rounds

Fig. S1

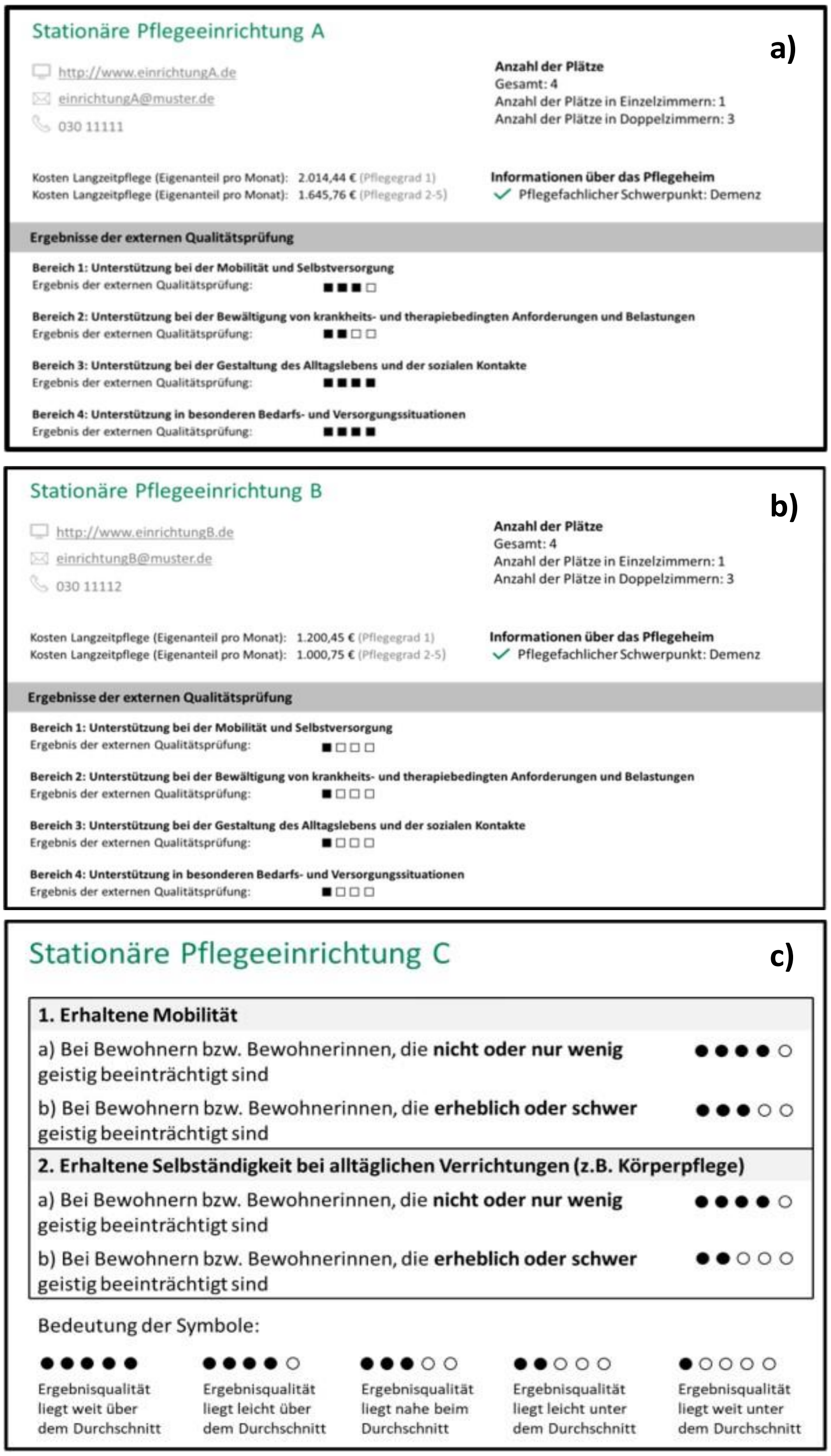

Format examples from three rounds of the comprehension test. (Source: own presentation modified according to AOK-Plegenavigator ; a) Identification of the best facility in round 1, b) Identification of the low-priced care in round 2; c) Identification of the above -average care for mobility in round 3)

DOI: 10.1007/s00391-024-02325-0

Fig. S2 Explanations for decision-making in comprehension test

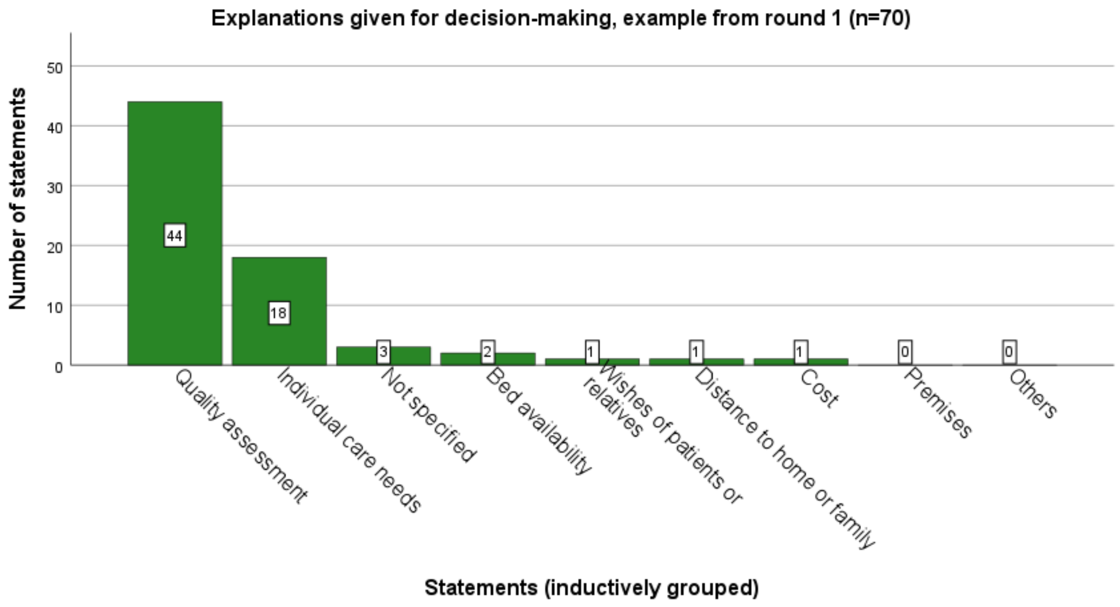

Fig. S3 Simplicity of the presentation format

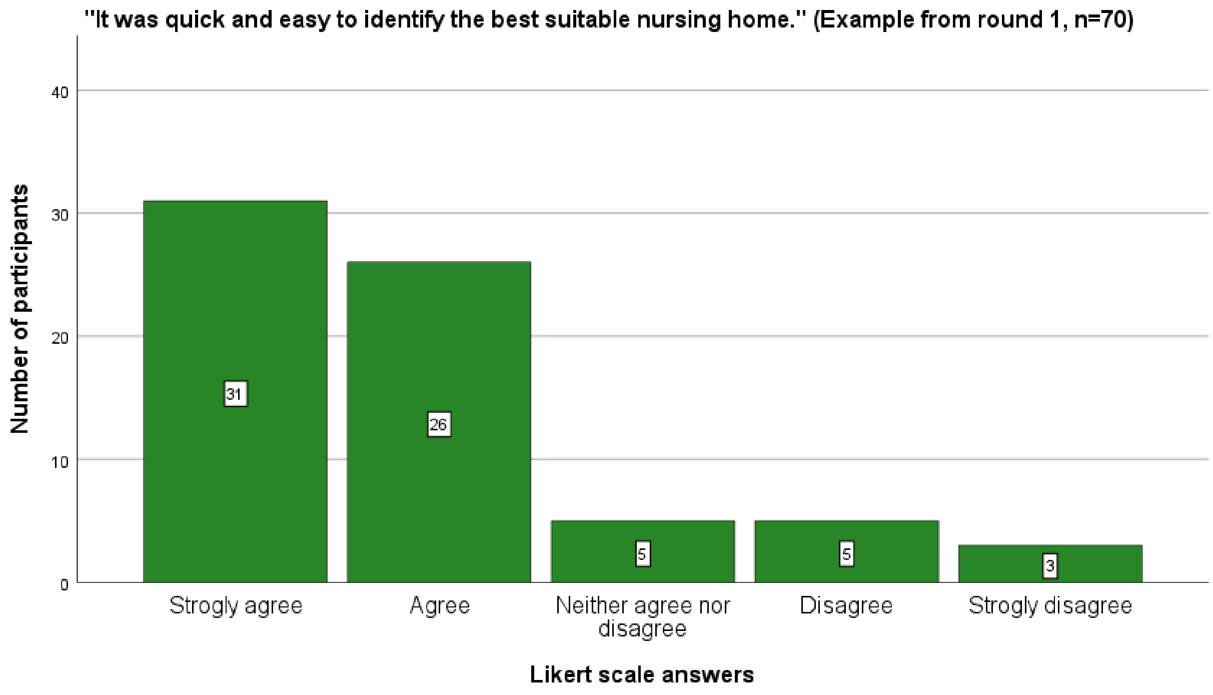

Supplementary material for the manuscript “**Transition from hospital to nursing home: Discharge planners as a potential lever for quality improvements?**”
Authors: Kristina Kast, Lukas Carl
DOI: 10.1007/s00391-024-02325-0

Fig. S4 Practicability of information presentation

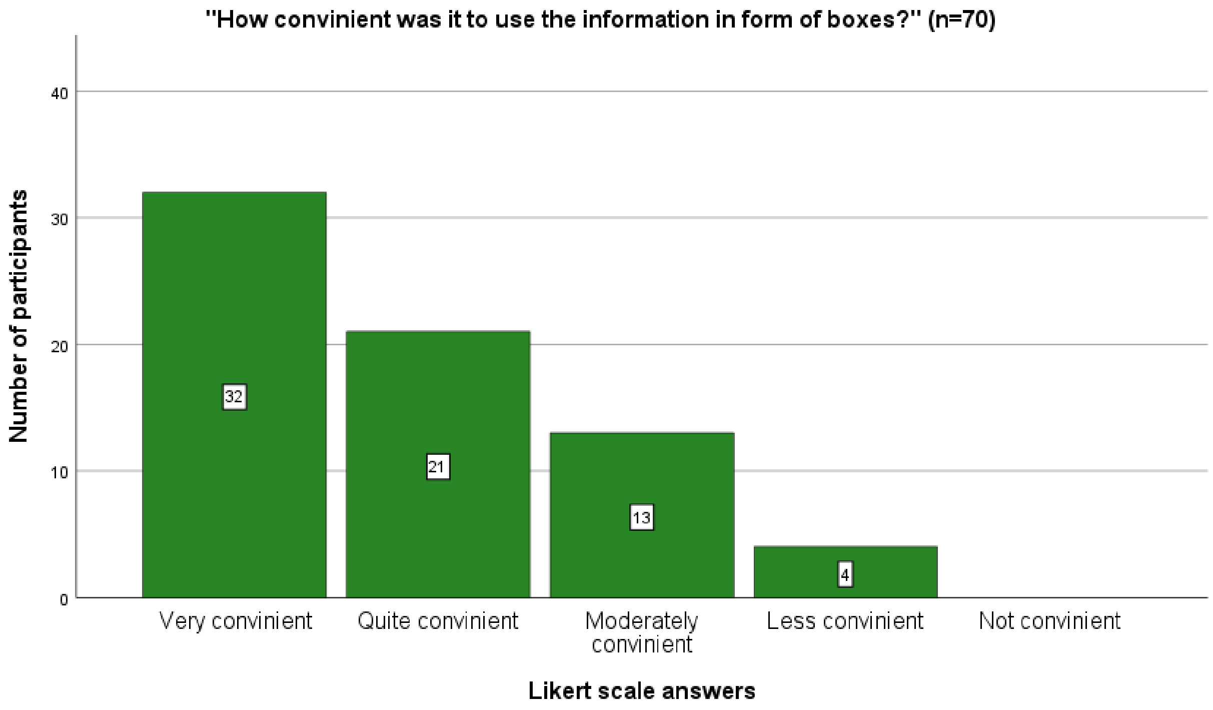

Supplementary material for the manuscript "**Transition from hospital to nursing home: Discharge planners as a potential lever for quality improvements?**"
Authors: Kristina Kast, Lukas Carl
DOI: 10.1007/s00391-024-02325-0

Table S1 Details on choice behavior of discharge planners

**Instruments used for search[§]**	**Count**	**%**	**Only one instrument**	**Combination of at least two instruments**	**p-value***
Internal nursing home directory of the hospital	62	92.5	9 (13.4)	53 (79.1)	<0.001
Public websites for nursing home search	43	64.2	3 (4.5)	40 (59.7)	
External platforms for discharge management	27	40.3	0 (0.0)	27 (40.3)	
Internal discharge management platform	13	19.4	0 (0.0)	13 (19.4)	
Other instruments[#]	5	7.5	0 (0.0)	5 (7.5)	
Total participants	67	100.00	12 (17.91)	55 (82.09)	
Total answers	150	-	12 (8.00)	138 (92.00)	
**Most important criteria for decision-making[§]**	**Count**	**%**	**Criterion 1 (%)**	**Criterion 2 (%)**	**Criterion 3 (%)**
Distance to home or family	55	28.80	22 (33.33)	23 (35.38)	10 (16.67)
Bed availability	51	26.70	17 (25.76)	18 (27.69)	16 (26.67)
Wishes of patients or relatives	41	21.47	20 (30.30)	9 (13.85)	12 (20.00)
Individual care needs	19	9.95	3 (4.55)	12 (18.46)	4 (6.67)
Organizational aspects	6	3.14	0 (0.0)	1 (1.54)	5 (8.33)
Financial aspects	5	2.62	0 (0.0)	1 (1.54)	4 (6.67)
Ownership	3	1.57	0 (0.0)	0 (0.0)	3 (5.00)
Combined criteria	3	1.57	2 (3.03)	0 (0.0)	1 (1.67)
Specializations	2	1.05	0 (0.0)	0 (0.0)	2 (3.33)
Quality assessment	1	0.52	1 (1.52)	0 (0.0)	0 (0.0)
Social compatibility	1	0.52	1 (1.52)	0 (0.0)	0 (0.0)
Rehabilitation needs	1	0.52	0 (0.0)	1 (1.54)	0 (0.0)
COVID-19 policies	1	0.52	0 (0.0)	0 (0.0)	1 (1.67)
Range of services	1	0.52	0 (0.0)	0 (0.0)	1 (1.67)
Premises	1	0.52	0 (0.0)	0 (0.0)	1 (1.67)
Valid answers	191	100.00	66 (100.00)	65 (100.00)	60 (100.00)
Not specified[$]	10	4.98[&]	1 (1.49)[&]	2 (2.99)[&]	7 (10.45)[&]
Total answers	201	-	67	67	67
**Involvement of patients or relatives in decision-making**	**Count**	**%**	**Mean**	**Median**	**p-value***
Always	28	41.79	1.78	2.00	<0.001
Often	28	41.79			
Sometimes	9	13.43			
Seldom	2	3.00			
Never	0	0.00			
Total participants	67	100	-	-	-

Data expressed as absolute numbers and percentages based on n=67 participants; [§]multiple answers were allowed; [#]other instruments: cooperation, care support centers, updates by nursing

DOI: 10.1007/s00391-024-02325-0

homes, phone calls, clinical network; *for "instruments": comparison of the group who uses only one instrument and the group who uses a combination of instruments, exact significance, two-tailed, calculated using binomial test; for "involvement": asymptotic significance of the two-sided one-sample chi-square test for testing if the levels of the variable occur with equal probabilities; [$]no clear statement provided or the same answer as in the previous category; [&]out of total answers

Table S2 Comprehension test results

**Comprehension by max. points**			
**Max. points**	**Achieved by number of participants (n=70)**	**Achieved by % of participants**	**Average points achieved (%)**
1	1	1.4	5.76 (82.24)
2	0	0.0	
3	2	2.9	
4	2	2.9	
5	12	17.1	
6	43	61.4	
7	10	14.3	
**Comprehension by task**			
**Task**	**Achieved points (%)[§]**	**Mistakes (%)**	**Complexity (p-value)**
Identification of the best facility (R1)	68 (97.1)	2 (2.9)	level 1 (0.000 [a)])
Identification of the best support with mobility and self-care (R1)	68 (97.1)	2 (2.9)	
Identification of the best facility (R2)	69 (98.6)	1 (1.4)	
Identification of the best facility (R3)	69 (98.6)	1 (1.4)	
Identification of the worst facility for the individual care needs (R1)	62 (88.6)	8 (11.4)	level 2 (0.000 [b)])
Identification of the low-priced care (R2)	56 (80.0)	14 (20.0)	
Identification of the above-average care for mobility (R3)	11 (15.7)	59 (84.3)	level 3 (0.000 [c)])
Total	403 (82.24)	87 (17.76)	

R1=round 1, R2=round 2, R3=round 3; [§]out of 70 possible points per task; p-values calculated using Friedman test with post hoc test, asymptotic significances (2-sided) are displayed, with Bonferroni correction; [a)]complexity level 1 vs. complexity levels 2 and 3, [b)]complexity level 2 vs. complexity levels 1 and 3, [c)]complexity level 3 vs. complexity levels 1 and 2
